# *Cronobacter sakazakii* ATCC 29544 Translocated Human Brain Microvascular Endothelial Cells via Endocytosis, Apoptosis Induction, and Disruption of Tight Junction

**DOI:** 10.3389/fmicb.2021.675020

**Published:** 2021-06-07

**Authors:** Tong Jin, Ning Guan, Yuhang Du, Xinpeng Zhang, Jiahui Li, Xiaodong Xia

**Affiliations:** ^1^College of Food Science and Engineering, Northwest A&F University, Xianyang, China; ^2^National Engineering Research Center of Seafood, Collaborative Innovation Center of Seafood Deep Processing, Dalian Polytechnic University, Dalian, China

**Keywords:** *Cronobacter sakazakii*, human brain microvascular endothelial cells, transcytosis, apoptosis, tight junction

## Abstract

*Cronobacter sakazakii* (*C. sakazakii*) is an emerging opportunistic foodborne pathogen that can cause neonatal necrotizing enterocolitis, meningitis, sepsis in neonates and infants with a relatively high mortality rate. Bacterial transcytosis across the human brain microvascular endothelial cells (HBMEC) is vital for *C. sakazakii* to induce neonatal meningitis. However, few studies focus on the mechanisms by which *C. sakazakii* translocates HBMEC. In this study, the translocation processes of *C. sakazakii* on HBMEC were explored. *C. sakazakii* strains could effectively adhere to, invade and intracellularly survive in HBMEC. The strain ATCC 29544 exhibited the highest translocation efficiency across HBMEC monolayer among four tested strains. Bacteria-contained intracellular endosomes were detected in *C. sakazakii*-infected HBMEC by a transmission electron microscope. Endocytosis-related proteins CD44, Rab5, Rab7, and LAMP2 were increased after infection, while the level of Cathepsin L did not change. *C. sakazakii* induced TLR4/NF-κB inflammatory signal pathway activation in HBMEC, with increased NO production and elevated mRNA levels of IL-8, IL-6, TNF-α, IL-1β, iNOS, and COX-2. *C. sakazakii* infection also caused LDH release, caspase-3 activation, and HBMEC apoptosis. Meanwhile, increased Dextran-FITC permeability and decreased trans epithelial electric resistance indicated that *C. sakazakii* disrupted tight junction of HBMEC monolayers, which was confirmed by the decreased levels of tight junction-related proteins ZO-1 and Occludin. These findings suggest that *C. sakazakii* induced intracellular bacterial endocytosis, stimulated inflammation and apoptosis, disrupted monolayer tight junction in HBMEC, which all together contribute to bacterial translocation.

## Introduction

*Cronobacter sakazakii* (*C. sakazakii*) is rod-shaped Gram-negative opportunistic pathogen, which is closely associated with neonatal necrotizing enterocolitis, sepsis, meningitis, and meningo-encephalitis in neonates and infants (Mullane et al., 2007; Weng et al., 2014). *C. sakazakii* could contaminate and survive in powdered infant formulas and infections in infants have been commonly associated with consumption of contaminated powdered infant formula (Hunter et al., 2008; Tall et al., 2014). Without appropriate treatment, *C. sakazakii* infection could lead to a relatively high mortality rate (approximately 33–80%) (Patrick et al., 2014; Lepuschitz et al., 2019; Elkhawaga et al., 2020).

*Cronobacter sakazakii* is one of the important pathogens implicated in neonatal meningitis (Ogrodzki and Forsythe, 2015). Previous studies have shown that several meningitis-related pathogens could penetrate the blood brain barrier (BBB) and invade the central nervous system (CNS). Once the pathogens enter the CNS, they could multiply and induce the release of proinflammatory cytokines and toxins (Sanders et al., 2011; Karassek et al., 2015), which leads to increased BBB permeability and the migration of lymphocytes, monocytes, and neutrophils across the BBB (pleocytosis), resulting in inflammatory responses in pallium tissues and meningitis (Mu et al., 2014). To cause infections in the brain, the pathogen needs to survive in the host bloodstream, cross the BBB and gain entry into the CNS (Van Sorge and Doran, 2012). *C. sakazakii* has been proved to be able to invade epithelial and endothelial cells and evade the immune responses, thereby leading to bacterial colonization and proliferation in the host CNS to induce meningitis (Almajed and Forsythe, 2016).

Blood brain barrier maintains the homeostasis of the CNS and is a critical line of defense against harmful microbial pathogens and other factors that potentially inflict damages on the host (Lemichez et al., 2010). BBB is mainly composed of endothelial cells and astrocytes lining the brain microvasculature (Abbott et al., 2010). Diverse factors facilitating the bacterial crossing of brain capillary endothelial cells have been identified, and several pathogens have been proved to cross BBB through both “transcellular” and “paracellular” routes (Neuhaus et al., 2018; Lu et al., 2019). Endocytosis plays an important role in translocation across BBB. For example, *Escherichia coli* K1 translocated HBMEC through endocytosis. The pathogen could adhere to receptors on the surface of HBMEC, stimulating the activation of the host molecules involved in a number of signaling pathways, including endocytosis-related proteins and kinases (Loh et al., 2017). A similar endocytosis process was also reported in Group B *Streptococcus* during the crossing of the BBB model (Benmimoun et al., 2020). Disruption of the BBB structure also facilitates the processes of bacterial translocation, and several inflammatory mediators (such as TNF-alpha, nitric oxide, and IL-6) contribute to HBMEC monolayer disruption (McLoughlin et al., 2017). Certain pathogens, like *Neisseria meningitidis*, could induce an increase of permeability in HBMEC, facilitating bacterial translocation across HBMEC monolayers (Schubert-Unkmeir et al., 2010). Some pathogens are proven to be able to disrupt endothelial tight junction structures by decreasing tight junction-related proteins (ZO-1, Occludin, and Claudin-5), which leads to an increase in bacterial translocation from the blood into CNS (Yuan et al., 2020).

Some studies have researched the mechanisms of *Cronobacter* pathogenesis in neonatal necrotizing enterocolitis, intestinal barrier translocation, and potential virulence factors (Liu Q. et al., 2012; Ye et al., 2016). For example, it has been proven that host cell actin rearrangements are required for *Cronobacter sakazakii* invasion into HBMEC (Li et al., 2010; Liu D. X. et al., 2012). However, the mechanisms by which *C. sakazakii* transverse HBMEC remain elusive.

This study aimed to decipher the routes through which *C. sakazakii* crosses HBMEC monolayers. Experiments were carried out to examine the bacterial endocytosis, inflammatory responses, endothelial monolayer permeability, cell apoptosis, and disruption of the tight junction in HBMEC infected by *C. sakazakii*.

## Materials and Methods

### Bacterial Strains and Cell Line

*Cronobacter sakazakii* strains (strain no. ATCC29544, ATCC12868, ATCC29004, and ATCCBAA-894) and human brain microvascular endothelial cells (HBMEC) were purchased from the American Type Culture Collection (ATCC). Bacterial strains were grown at 37°C in brain heart infusion (BHI) broth (Landbridge, Beijing, China) overnight. HBMEC were grown in DMEM (Dulbecco’s modified eagle medium, Gibco, Grand Island, NY, United States) with 10% FBS (Biological Industries, Kibbutz Beit Haemek, Israel) at 37°C and 5% CO_2_ for the indicated period of time in different experiments.

### Bacterial Adhesion to HBMEC

Adhesion assays were performed as previously described with some modifications (Amalaradjou et al., 2014). Briefly, HBMEC were seeded into 24-well plates (10^5^ cells per well), grown in DMEM (Dulbecco’s modified eagle medium) with 10% FBS at 37°C and 5% CO_2_ for 18 h (70–90% confluence in the well), and then rinsed twice with PBS (Beyotime, Shanghai, China). *C. sakazakii* strains (ATCC29544, 12868, 29004, BAA-894) were cultured in BHI broth at 37°C overnight. The bacterial suspensions were washed twice with PBS and resuspended in DMEM to approximately 10^7^ CFU/mL. Then 1ml of *C. sakazakii* suspension was added to each well at a multiplicity of infection (MOI) of 100:1 and incubated at 37°C in a humidified 5% CO_2_ incubator for 2 h. After incubation, the infected monolayers of HBMEC were washed twice with ice-cold PBS and lysed with 1 mL 0.1% Triton X-100 (Beyotime) at 4°C for 25 min. The lysates were serially diluted, plated onto LB agar (Landbridge), and incubated at 37°C overnight for counting.

### Invasion and Intracellular Survival

The invasion and intracellular survival were examined as previously described with minor modifications (Ryan et al., 2018). HBMEC and *C. sakazakii* samples were prepared as in the adhesion assay. After incubation with bacteria for 2 h, HBMEC monolayers were washed once with PBS and incubated for 40 min with DMEM containing gentamicin (100 μg/mL) to kill the extracellular bacteria. HBMEC were then washed and cultured with DMEM containing 10 μg/mL gentamicin for an additional 0, 2, 4, and 6 h. After being washed with PBS three times, HBMEC monolayers were lysed with 0.1% Triton X-100 at 4°C for 25 min. The sample dilutions were then plated on LB agar to count the invaded bacterial (0 h) and intracellularly survived bacteria (2, 4, and 6 h).

### Transcytosis Assay

**FIGURE 1:**
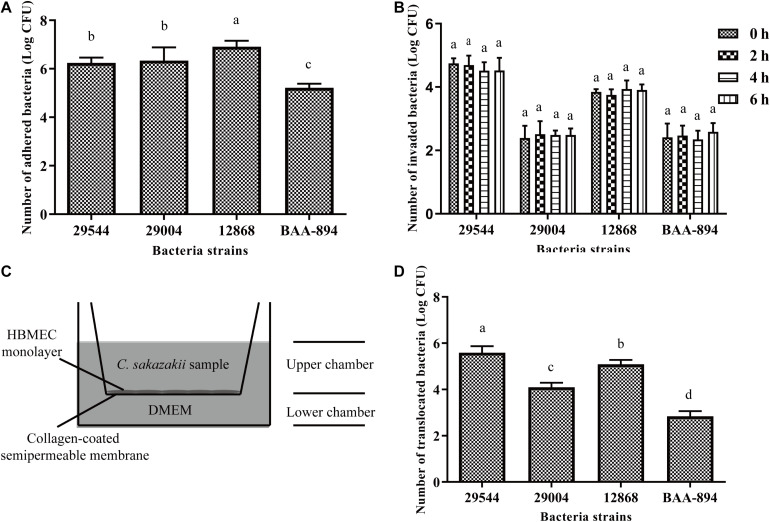
Adhesion, invasion, intracellular replication, and translocation of *C. sakazakii* in HBMEC. **(A)** The number of *C. sakazakii* cells that adhere to HBMEC. **(B)** The number of *C. sakazakii* cells in HBMEC after 2 h invasion and intracellular bacteria counts at 2, 4, and 6 h post-invasion. **(C)** HBMEC were incubated in transwell inserts. **(D)** The counts of *C. sakazakii* that translocated HBMEC monolayers were calculated. The strain ATCC 29544 performed the strongest invasion and translocation abilities compared to other strains (*P* < 0.01). Bars represent the means ± standard deviations (*n* = 3). Mean values with different lower-case letters are statistically different (*P* < 0.05).

The transwell systems were employed for transcytosis assay as previously described (Chen et al., 2019), HBMEC were seeded (2 × 10^4^ cells/insert) onto the inner surface of collagen-coated transwell inserts (24 well format, 3.0-μm pore size polycarbonate filter; Corning, NY, United States) which were placed in wells of a 24-well plate (Figure 1C). After growth in DMEM (containing 10% FBS) for 5 days, the HBMEC monolayers formed tight junctions, which was evidenced by TEER measurement. After washing with PBS three times, each transwell was moved to a new well containing 800 μL of fresh FBS-free DMEM medium, and the top well was replaced with 500 μL of *C. sakazakii* suspensions. The HBMEC monolayer was incubated at 37°C under 5% CO_2_ for 3 h, then the samples from the bottom well were serial diluted and plated on LB agar and cultured at 37°C overnight to count the number of translocated *C. sakazakii*.

### Transmission Electron Microscope (TEM)

HBMEC were seeded into a 90-mm culture dish (10^5^ per dish) and incubated at 37°C, 5% CO_2_ overnight to 70–90% confluence. Then HBMEC were infected with *C. sakazakii* ATCC 29544 for 0, 1, 2, and 3 h (MOI = 100). TEM samples were prepared as previously described (Lu et al., 2019). In brief, the infected cells were fixed with 2.5% glutaraldehyde for 6 h, followed by treatment with 1% osmic acid buffer for 2 h. Fixed HBMEC were dehydrated using an ethanol gradient (30, 50, 70, 80, 90, and 100%), then embedded and polymerized with the LR white with resin gradient to achieve final ultrathin sections (70 nm) on an ultramicrotome (UCT, Leica Microsystems). Ultrathin sections were mounted onto copper grids and stained with 4% uranyl acetate and lead citrate. Imaging was performed using a Transmission Electron Microscope (FEI TECNAI G2 SPIRIT BIO) operating at 120 kV. Digital images of HBMEC were captured using the Quemesa Bottom-Mount CCD and Morada G3 Side-Mount CCD Camera.

### Immunofluorescence

Immunofluorescence assays were performed according to a previous study with some modifications (Mu et al., 2014). For bacterial visualization, a GFP-labeled plasmid pCA66-GFP was electrotransformated into *C. sakazakii* ATCC 29544. The plasmid did not affect the invasion abilities of the strain (data not shown). HBMEC were propagated on round glass coverslips within 24-well plates to 70–90% confluence and infected with GFP-labeled *C. sakazakii* (MOI = 100). After infection for 0, 1, 2, 3, and 4 h, the cells were washed with PBS and fixed with 4% paraformaldehyde at 4°C for 1 h. After blocking with QuickBlock^TM^ Blocking Buffer (Beyotime, Shanghai, China) for 20 min at room temperature, the samples were incubated with the 1:100 dilutions of primary antibodies of CD44 (Proteintech, Wuhan, China), Rab5 (Beyotime), Rab7 (Beyotime), LAMP2 (Beyotime), Cathepsin L (Proteintech), and NF-κB p65 (Proteintech) at 4°C overnight. Cy-3 labeled secondary antibodies were used at a 1:500 dilution at room temperature. Samples on the slides were washed with PBS and mounted onto microscope slides with antifade reagent with DAPI (Beyotime). The images of protein expression and distribution in HBMEC were obtained with confocal laser scanning microscopy (Revolution-XD, Andor, England).

### Western Blot

HBMEC were prepared as in TEM assay (for detection of tight junction related-proteins ZO-1 and Occludin, HBMEC were grown for 5 days to form tight junction) and then infected with *C. sakazakii* ATCC 29544 for 0, 1, 2, 3, and 4 h (MOI = 100). Then, the samples were washed twice with ice-cold PBS and the cell lysates were prepared in lysis buffer with proteinase inhibitor cocktails (Beyotime). The supernatants were centrifuged at 13000 × *g* for 10 min at 4°C, and the protein concentrations were measured with a Bradford assay kit (Beyotime). An equal amount of proteins (30 μg) from samples were resolved by SDS-polyacrylamide gel electrophoresis and electrotransferred onto a nitrocellulose (NC) membrane. After being blocked, the NC membrane was subsequently incubated with specific primary antibodies at 4°C overnight and then washed by TBST (Tris-buffered saline, Tween 20, Beyotime). Horseradish peroxidase-conjugated secondary antibodies (1:1000 in TBST) were added and the mixture was incubated for 90 min at room temperature. After washing, the blots were detected with ECL reagents (Beyotime) and ChemiDocXRS+System (Bio-Rad, CA, United States). ACTB (β-actin) was used as an internal standard, the expression levels of CD44, Cathepsin L, TLR4, IκBα, ZO-1, Occludin, Rab5, Rab7, and LAMP2 were detected.

### Quantitative RT-qPCR

HBMEC were seeded in 60-mm dishes to 70–90% confluence and infected with *C. sakazakii* ATCC 29544 for 0, 1, 2, 3, and 4 h (MOI = 100). Total RNA was extracted from HBMEC using RNAeasy^TM^ Animal RNA Isolation Kit R0026 (Beyotime), and then reverse transcribed to cDNA using Evo M-MLV RT Premix AG11706 (Accurate Biotechnology, Changsha, China) with the suppliers’ instructions. Quantitative Real-Time PCR was performed with SYBR^®^ Green Premix Pro Taq HS qPCR Kit AG11701 (AG11701; Accurate Biotechnology) on a Bio-Rad iQ5 PCR system (Bio-Rad). The primer sequences for RT-PCR are shown in Table 1. ACTB was used as the control. The Ct values for RT-PCR samples were recorded. The relative mRNA levels were analyzed with the 2^−ΔΔCt^ method.

**TABLE 1:** Primer sequences used in RT-qPCR.

Target gene	Primer sequences (5′-3′)	References
*IL-8*	F GAGATAATGCACCCCGGACC R TGTTCTCACAGGAGAGAGTTGA	Wu et al., 2020
*iNOS*	F AGGGATTTTAACTTGCAGGTCC R AGGAGCCGTAATATTGGTTGACA	Wu et al., 2020
*TNF-α*	F GGCAGTCAGATCATCTTCTCGAAC R TGGTAGGAGACGGCGATGC	
*IL-1β*	F AGCTGGAGAGTGTAGATCCCAA R TGTTTTCTGCTTGAGAGGTGCT	Wu et al., 2020
*IL-6*	F TTCAATGAGGAGACTTGCCTG R ACAACAACAATCTGAGGTGCC	Brozek et al., 2008
*COX-2*	F TCCACAACCCTCTGCAC R TGCATTCTTTGCCCAGCACT	Kaulmann et al., 2016
*ACTB*	F ATCTGGCACCACACCTTCTACAATGAGCTGCG R CGTCATACTCCTGCTTGCTGATCCACATCTGC	

### Nitric Oxide Assay

To detect nitric oxide (NO) release from HBMEC, the cells were seeded into a 96-well plate (10^4^ cells/well) and grown overnight to 70–90% confluence. Then cells were infected with *C. sakazakii* ATCC 29544 for 0, 1, 2, 3, and 4 h (MOI = 100). NO concentrations were quantified using a nitric oxide assay kit S0021S (Beyotime). Briefly, 50 μL of Griess reagent I and Griess reagent II were dripped into each well containing 50 μL of culture supernatants or NaNO_2_ standards. After reaction for 5 min, the absorbance of samples was measured at 540 nm utilizing a microplate reader (Victor X3; PE, Singapore) to determine the NO release.

### Lactate Dehydrogenase (LDH) Release

The procedures for *C. sakazakii* infection of HBMEC were the same as the NO release assay. LDH release was detected using an LDH Cytotoxicity Assay Kit C0017 (Beyotime). The absorbance at 490 nm of samples was measured with a microplate reader (Victor X3; PE, Singapore) to evaluate LDH release after C. sakazakii infection. The percentage of LDH release was calculated as follows: % LDH release = (sample LDH activity-background LDH)/(total LDH activity-background LDH) ×100.

### Caspase-3 Activity and Apoptosis Assay

HBMEC cells were seeded into a 60-mm dish and grown overnight to 70–90% confluence. Then cells were infected with *C. sakazakii* for 0, 1, 2, 3, and 4 h to detect Caspase-3 activity and apoptosis levels. Caspase-3 activity was detected with a Caspase-3 Activity Assay Kit C1115 (Beyotime). In brief, cells were lysed on an ice bath and centrifuged (18000 *× g*, 15 min, 4°C) to collect the supernatant. After incubation with Ac-DEVD-*p*NA for 20 h at 37°C, the samples were measured with a microplate reader (VictorX3, PE, Singapore) at an absorbance of 405 nm.

Apoptosis assay was performed with Annexin V-FITC/PI Apoptosis Detection Kit C1062S (Beyotime) according to the instructions. HBMEC were collected and washed twice with PBS after treatments, then the cells were stained with Annexin V-FITC and PI reagents. Cell apoptosis levels were measured with a flow cytometer (FACSAria TM III, BD, New Jersey, United States).

### Trans Epithelial Electric Resistance (TEER) and Permeability of HBMEC Monolayers

HBMEC were cultured using a transwell system to form a tight junction as previously described. *C. sakazakii* suspension was added into the inner side of the transwell inserts and incubated for 0, 1, 2, 3, and 4 h. After treatments, the integrity of the HBMEC cellular monolayer was monitored for TEER using a MillicellR ERS-2 meter (Millipore, United States). The permeabilities of HBMEC monolayers were measured by the transport of 4 kDa Dextran-FITC (FD4; Sigma-Aldrich, St. Louis, MO, United States). Dextran-FITC was dissolved in FBS-free DMEM (1 mg/mL) and added to the PBS-washed apical surface of the transwell insert (400 μL per insert) after *C. sakazakii* infection, 800 μL of pure DMEM medium was added to the bottom side of the transwell systems. After 4 h incubation, fluorescence intensities of the medium from the transwell bottom sides were measured using a fluorescence microplate reader (485 nm excitation/520 nm emission).

### Statistical Analysis

All experiments were performed in triplicate. Statistical analyses were performed in SPSS software (Version 22.0; SPSS, Inc., Chicago, IL, United States). The data are presented as the mean ± SD and differences between means were tested by one-way ANOVA. The *p* values < 0.05 were considered to be statistically significant.

## Results

### *C. sakazakii* Invaded, Survived in, and Translocated Across HBMEC

As shown in Figure 1, all four tested *C. sakazakii* strains (ATCC 29544, 29004, 12868, BAA-894) could adhere to (A) and invade (B) HBMEC cells. After *C. sakazakii* strains invasion, the number of intracellular survived *C. sakazakii* bacteria in HBMEC did not change significantly after 6 h (*P* > 0.1), which indicates intracellular survival without replication in HBMEC (Figure 1B). Four strains were also efficiently translocated across the HBMEC monolayers (Figure 1D). Among four strains, *C. sakazakii* ATCC 29544 was the most efficient in invading and translocating across HBMEC. Therefore, only the strain ATCC 29544 was used for further studies.

### Endocytosis of *C. sakazakii* in HBMEC

**FIGURE 2:**
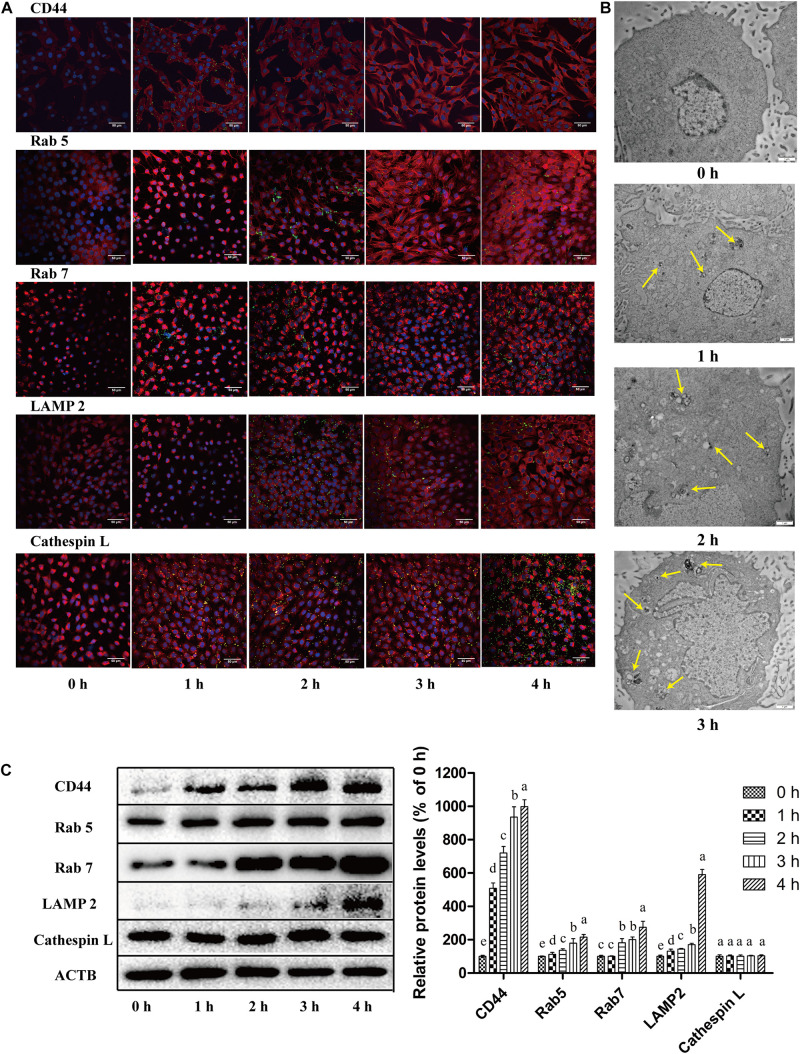
Bacterial endocytosis and intracellular transport of *C. sakazakii* in HBMEC after the invasion. **(A)** The expression and localization of endocytosis-related proteins (CD44, Rab5, Rab7, LAMP2, and Cathepsin L) (red fluorescence) in HBMEC (DPAI, blue fluorescence for cell nucleus) infected with *C. sakazakii* (labeled with GFP, green fluorescence) were analyzed with immunofluorescent staining and observed with a confocal laser scanning microscope. **(B)** The *C. sakazakii*-containing endocytosis vacuoles (yellow arrows) were examined in HBMEC using transmission electron microscopy after infection. **(C)** The expression levels of CD44, Rab5, Rab7, LAMP2, and Cathepsin L in *C. sakazakii*-infected HBMEC were detected with Western Blot using ACTB as a control. Representative blots for proteins (left) and quantitative analysis (right) were presented. Bars represent the means ± standard deviations (*n* = 3). Mean values with different lower-case letters are statistically different (*P* < 0.05).

*Cronobacter sakazakii* bacteria were found in vacuoles of the HBMEC by transmission electron microscopy (Figure 2B). From 1 h to 3 h post-infection, the presence of intracellular endosomes containing bacteria increased in HBMEC, presumably indicating that the strain is undergoing transcytosis.

To confirm the transport of *C. sakazakii* through endosomes, endocytosis-related proteins were measured with immunofluorescence (Figure 2A) and western blot (Figure 2C). Cell-surface glycoprotein CD44 (an adhesion receptor) was labeled red under fluorescence and its expression in infected cells was increased by 9 fold at 4 h post-infection compared to uninfected HBMEC. Expression of early (Rab5) and late (Rab7) endosomal markers also increased significantly in infected HBMEC (*P* < 0.05). *C. sakazakii*-containing intracellular vacuoles colocalized with Rab5 and Rab7. The expression of a lysosomal marker, LAMP2, which facilitates the fusion of intracellular vacuoles with lysosomes, was also enhanced in HBMEC after *C. sakazakii* infection. Additionally, *C. sakazakii* infection did not change the levels of the lysosomal enzyme Cathepsin L. Meanwhile, co-localization rates of GFP-labeled *C. sakazakii* with endocytosis-related proteins in HBMEC were analyzed with immunofluorescence (Table 2). Intracellular bacteria are closely associated with CD44, Rab5, Rab7, and LAMP2. However, intracellular *C. sakazakii* showed a limited association with Cathepsin L. Taken together, intracellular *C. sakazakii* ATCC 29544 is associated with endocytosis, which could contribute to bacterial translocation across HBMEC.

**TABLE 2:** Co-localization rates of GFP-labeled *C. sakazakii* with endocytosis-related proteins in HBMEC.

Proteins	Co-localization rates (%)			
	1 h	2 h	3 h	4 h
CD44	82.59 ± 4.47*^*b*^*	88.04 ± 2.61*^*a*^*	88.96 ± 1.45*^*a*^*	88.82 ± 1.07*^*a*^*
Rab5	86.12 ± 6.17*^*a*^*	86.17 ± 1.09*^*a*^*	88.17 ± 1.01*^*a*^*	90.06 ± 2.28*^*a*^*
Rab7	90.10 ± 2.71*^*a*^*	90.63 ± 1.88*^*a*^*	90.87 ± 1.31*^*a*^*	91.01 ± 1.77*^*a*^*
LAMP2	88.99 ± 1.93*^*a*^*	89.54 ± 0.62*^*a*^*	90.06 ± 0.60*^*a*^*	90.23 ± 0.90*^*a*^*
Cathepsin L	27.84 ± 3.12*^*a*^*	31.09 ± 5.83*^*a*^*	31.32 ± 7.73*^*a*^*	33.11 ± 3.29*^*a*^*

*Bars represents the standard deviation (*n* = 3), mean values with different lower-case letters are statistically different (*P* < 0.05).*

### *C. sakazakii* Induced Inflammatory Responses in HBMEC

**FIGURE 3:**
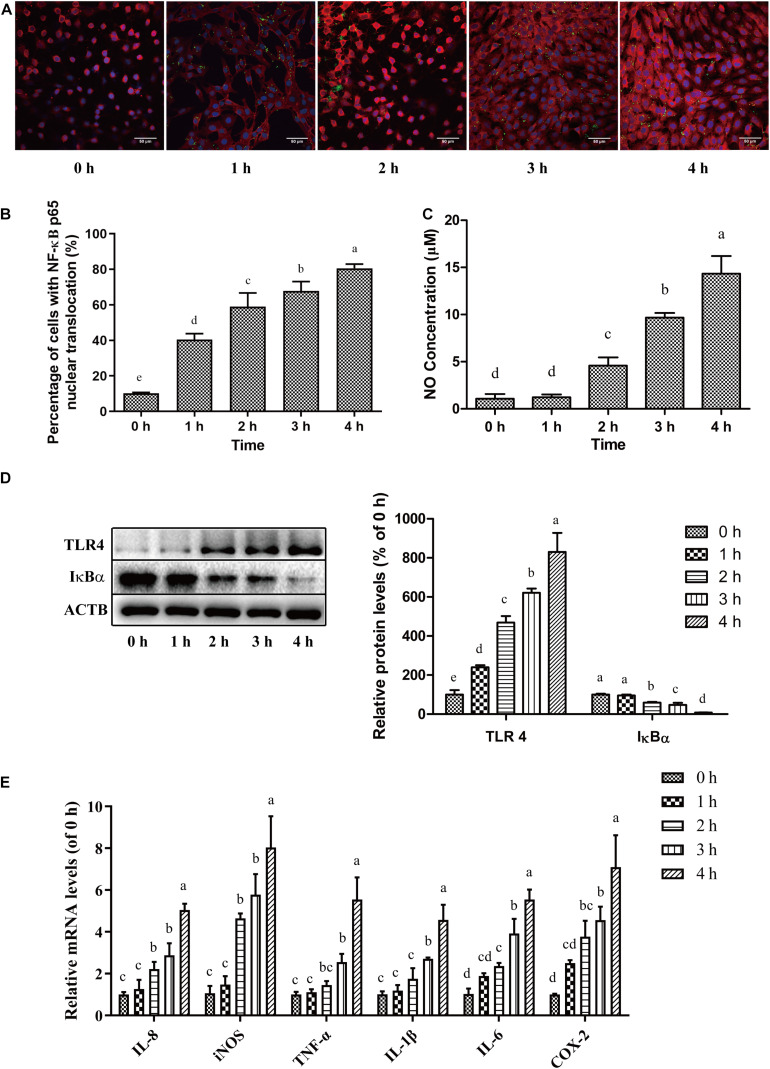
*C. sakazakii* infection induced inflammatory responses in HBMEC. After *C. sakazakii* infection, the nuclear translocation of NF-κB p65 in HBMEC was analyzed with immunofluorescent staining. **(A)** Representative fluorescence micrograph and **(B)** quantitative analysis were presented. **(C)** The concentrations of NO in culture medium supernatant was measured. **(D)** The blots (left) and quantitative analysis (right) for IκBα and TLR4 protein in HBMEC after *C. sakazakii* infection. **(E)** Relative mRNA levels of IL-8, IL-6, TNF-α, IL-1β, iNOS, and COX-2 in HBMEC after *C. sakazakii* infection for 0, 1, 2, 3, and 4 h. Error bars represent the standard deviation (*n* = 3). Mean values with different lower-case letters are statistically different from one another (*P* < 0.05).

As shown in Figure 3A, *C. sakazakii* infection stimulated the translocation of NF-κB p65 from the cytoplasm to the nucleus in HBMEC. After infection for 4 h, 80.63% of HBMEC were positive for NF-κB p65 nuclear translocation (Figure 3B). To further demonstrate the activation of the inflammatory pathway, TLR4/NF-κB pathway related proteins were detected by western blot (Figure 3D). After infection for 4 h, relative expression levels of TLR4 in HBMEC increased 8.30 fold. Meanwhile, a significant reduction was observed for IκBα expression, contributing to the activation of the TLR4/NF-κB inflammation pathway.

Nitric oxide (NO), a cellular inflammatory response product, was also detected (Figure 3C). After *C. sakazakii* infection for 0, 1, 2, 3, and 4 h, the concentrations of NO were 1.08 ± 0.51, 1.22 ± 0.30, 4.59 ± 0.87, 9.68 ± 0.50, and 14.34 ± 1.87 μM, respectively. The expressions of inflammation-related genes were shown in Figure 3E. *C. sakazakii* infection up-regulated the transcriptional levels of 6 inflammation-associated genes. Exposure to *C. sakazakii* increased the mRNA expressions of cytokines such as IL-8, IL-6, TNF-α, IL-1β, and proinflammatory factors such as iNOS, COX-2 in HBMEC in a time-dependent manner.

### Cytotoxic and Apoptotic Effect of *C. sakazakii* on HBMEC

**FIGURE 4:**
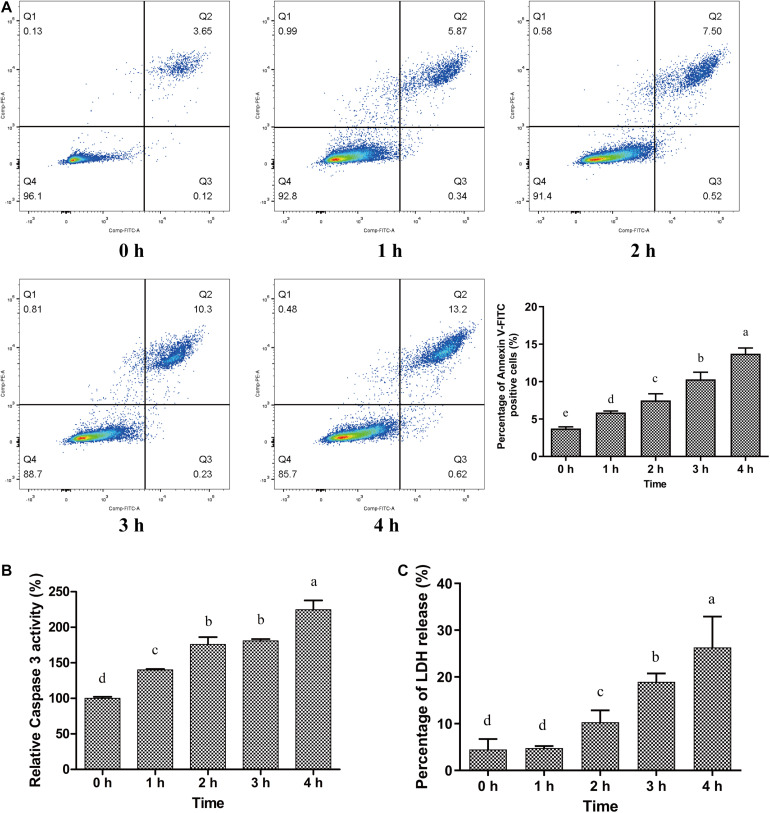
Cytotoxic and apoptotic effect of *C. sakazakii* on HBMEC. **(A)** *C. sakazakii*-infected HBMEC were stained with Annexin V-FITC/PI and analyzed with a flow cytometer. **(B)** Caspase-3 activity in HBMEC after *C. sakazakii* infection for 0, 1, 2, 3, and 4 h. **(C)** Relative LDH release from *C. sakazakii*–infected HBMEC. Error bars represent the standard deviation (*n* = 3), Mean values with different lower-case letters are statistically different from one another (*P* < 0.05).

After *C. sakazakii* infection, lactate dehydrogenase (LDH) was rapidly released into the cell culture supernatant from HBMEC (Figure 4C), relative LDH release increased to 29.77% after 4 h-infection, indicating damage in the cell plasma membrane. To investigate whether *C. sakazakii* infection induces the cytotoxic and apoptotic effect in HBMEC, caspase-3 activity and apoptosis levels were measured. The activity of caspase-3 in *C. sakazakii*-induced HBMEC was shown in Figure 4B. Compared to control HBMEC, caspase-3 activity in HBMEC infected with *C. sakazakii* significantly increased by 145.45% (*P* < 0.05) after 4 h.

The apoptotic HBMEC were stained with Annexin V/FITC and PI and analyzed using a flow cytometer. As shown in Figure 4A, only 3.74% of the control HBMEC were stained with Annexin V/FITC. After *C. sakazakii* infection for 1, 2, 3, and 4 h, the percentage of Annexin V/FITC positive HBMEC significantly increased to 5.88, 7.29, 10.31, and 13.73%, respectively (*P* < 0.05). Interestingly, nearly all Annexin V/FITC positive HBMEC were also stained with PI, which indicated that *C. sakazakii* induced late-stage apoptosis in HBMEC. Altogether, *C. sakazakii* strain infection caused cytotoxicity and apoptosis in HBMEC.

### *C. sakazakii* Disrupted Tight Junction of HBMEC Monolayers

**FIGURE 5:**
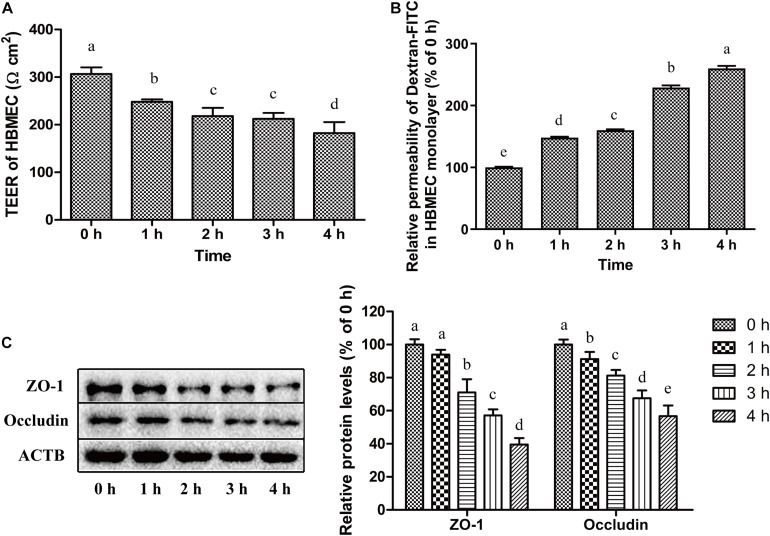
*C. sakazakii* disrupted tight junction of HBMEC monolayers. **(A)** The TEER of HBMEC monolayer after *C. sakazakii* infection for 0, 1, 2, 3, and 4 h. **(B)** Dextran-FITC (4 kDa) permeability in HBMEC monolayer infected with *C. sakazakii*. **(C)** The expression of tight junction-related proteins ZO-1 and Occludin in *C. sakazakii*-infected HBMEC were detected by Western Blot. Error bars represent the standard deviation (*n* = 3). Mean values with different lower-case letters are statistically different from one another (*P* < 0.05).

Trans-epithelial electrical resistance (TEER) of HBMEC monolayers (on a transwell system) before and after *C. sakazakii* infection are presented in Figure 5A. HBMEC monolayers without *C. sakazakii* infection demonstrated a stable TEER of around 306.75 ± 13.72 Ω cm^2^, which indicates the formation of a monolayer tight junction after 5 days of growth. After being challenged with *C. sakazakii* for 1–4 h, TEER of monolayers was reduced in a time-dependent manner and decreased to 182.60 ± 22.80 Ω cm^2^ after 4 h-infection (*P* < 0.05). The permeabilities of HBMEC monolayers, calculated based on the Dextran-FITC (4 kDa) transport, are presented in Figure 5B. Dextran permeability increased in a time-dependent manner after infection. After 4 h exposure to *C. sakazakii*, dextran permeability of HBMEC monolayers increased by 1.6 folds compared to control cells. In addition, protein expression of tight junction-related proteins ZO-1 and Occludin (Figure 5C) were significantly reduced in *C. sakazakii* infected cells. The relative expression of ZO-1 and Occludin significantly decreased to 39.54% and 56.73% of the control, respectively, in HBMEC after infection for 4 h (*P* < 0.05).

## Discussion

*Cronobacter sakazakii-*contaminated powdered infant formulas have been associated with outbreaks of infant meningitis (Alzahrani et al., 2015). To cause meningitic infections, *C. sakazakii* is required to cross the blood brain barrier and gain access to the CNS (Townsend et al., 2007). Mange et al. (2006) showed that *C. sakazakii* isolates effectively adhered to the HEp-2 cells, Caco-2 cells, and HBMEC. In this study, four *C. sakazakii* strains were shown to not only adhere to and invaded HBMEC, but also traverse across HBMEC. Similarly, *Escherichia coli* K1 has been demonstrated to invade and translocate HBMEC monolayers (Zhang et al., 2002). It has also been proved that transcytosis is important for *Streptococcus pneumoniae* to cause bacteremia and meningitis (Brissac and Orihuela, 2019). Bacterial intracellular replication also plays an important role in some bacterial infections. For example, *Citrobacter freundii* and *Neisseria meningitidis* strains could survive intracellularly and replicate in HBMEC when translocating the monolayer (Badger et al., 1999; Nikulin et al., 2006). However, we observed that the number of intracellular *C. sakazakii* bacteria did not increase or reduce significantly in HBMEC during translocation.

Mehta et al. (2006) also reported that *Mycobacterium tuberculosis* could enter, internalize and translocate across human microvascular endothelial cells, but lack the ability to replicate intracellularly. Thus, the translocation of *C. sakazakii* in HBMEC seems not to be dependent on intracellular replication. Interestingly, *C. sakazakii* could survive intracellularly and replicate in macrophage cells (Shi et al.,2017a,b; Kim S. et al., 2017), and the immune-related pericytes and leucocytes are also essential for the stabilization of BBB. Further research will focus on the role of *C. sakazakii* intracellular survival in HBMEC monolayer during bacterial translocation.

Bacterial endocytosis and intracellular transport are important for certain pathogens to penetrate and cross the HBMEC monolayer. Loh et al. (2017) reported that *Escherichia coli* K1 utilized host macro-pinocytic pathways and intracellular vesicles for transcytosis in HBMEC. Using TEM, we discovered the presence of *C. sakazakii*–containing vacuoles in HBMEC after infection. Similarly, Giri et al. (2012) also demonstrated intracellular *Cronobacter* within host vacuoles in HBMEC during translocation. We discovered that *C. sakazakii* stimulated the expression of the transmembrane glycoprotein CD44 (an adhesion receptor for pathogen) in HBMEC, indicating the transmembrane invasion and transport of the pathogen. Similarly, a previous study showed that *Shigella* virulence proteins bound to CD44 on host cytomembrane for bacterial invasion (Skoudy et al., 2000).

*Cronobacter sakazakii* infection up-regulated the levels of endocytotic vesicle formation and maturation marker proteins Rab5 and Rab7 in HBMEC. It has been proved that Rab5 and Rab7 positively contributed to bacterial invasion into host cells (Mottola et al., 2014). In this study, it was shown that intracellular *C. sakazakii* existed in the Rab5 and Rab7 positive vacuole compartment. Previous studies have shown that rab5 and rab7 played an essential role in the trafficking of *E. coli*-containing vacuoles and intracellular bacterial survival in HBMEC (Kim et al., 2003; Mu et al., 2016). It can be inferred that endocytosis is an important strategy for *C. sakazakii* to translocate across HBMEC.

After *C. sakazakii* infection, lysosomal marker LAMP2 levels increased and relocalized, indicating that endosomes containing *C. sakazakii* mature into endolysosomes. The previous study demonstrated that *Trypanosoma cruzi* invasion into human epithelial HeLa cells was accomplished through recognition of gp82 by its receptor LAMP2 protein (Rodrigues et al., 2019). Kuhbacher et al. (2018) showed that when *Listeria monocytogenes* invaded epithelial cells, the lysosomal-associated membrane proteins LAMP1 and LAMP2 are translocated to the cellular surface, and the activation of several signaling pathways promoted escape of bacteria from endosomes and transcytosis of *L. monocytogenes*. As late endosomes/lysosomes are the major donor compartments of LAMP2, these organelles are likely to participate in *C. sakazakii* invasion and translocation in HBMEC. The lysosomal enzyme Cathepsin L is involved in cellular invasion and vesicle degradation (Xiong et al., 2017). Cathepsin L was activated in swine dendritic cells (DCs) and B cells after *Mycoplasma hyopneumoniae* infection, contributing to the inflammatory responses induced by pathogens (Zhang et al., 2019). In this work, the Cathepsin L in HBMEC were not activated by *C. sakazakii*. Similarly, *Helicobacter pylori* infection caused inflammation in gastric mucosa, but Cathepsin L expression in gastric mucosa epithelial cells was still at very low levels during *Helicobacter pylori* infection (Buhling et al., 2004). Pathogen-induced inflammatory signaling pathway activation and cytokines expressions in BBB-related cells mediated its intracellular transcytosis processes (Miller et al., 2005). We showed that *C. sakazakii* could stimulate NF-κB p65 nuclear translocation and TLR4 expression, suggesting NF-κB inflammation pathway activation. Some pathogens, like *Neisseria meningitidis* and *Streptococcus pneumoniae* could express microbial-associated molecular patterns, which are recognized by pattern recognition receptors (such as TLR4) on the cell surface. The binding recruits a cohort of signaling molecules and drives signaling pathway activation, which promotes bacterial translocation (Telleria-Orriols et al., 2014). It could be inferred that, once *C. sakazakii* interacts with TLR4, inflammation-related proteins were phosphorylated and disassociated, activating the NF-κB pathway and inducing nitric oxide release. NO regulated cellular inflammation and permeability.

A previous study has identified that lipoteichoic acid in Gram-positive pathogens activated glial cells to produce nitric oxide and pro-inflammatory cytokines, which contributed to the disruption of BBB structure and function in a concentration- and time-dependent manner (Boveri et al., 2006). An increase in the transcription of IL-8, IL-6, TNF-α, IL-1β, iNOS, and COX-2- mRNA was also noted. Previous studies showed that cytokines IL-6, TNF-α, and IL-1β and some other inflammatory factors regulated HBMEC apoptosis or monolayer tight junction disruption, and increased BBB permeability (Wong et al., 2004; Poller et al., 2010; Barichello et al., 2011). We observed that *C. sakazakii* induced inflammatory responses in HBMEC, and proinflammatory cytokines releases are likely to facilitate disruption of HBMEC monolayers integrity. It was reported that the different types of bacteria could interact with host BMEC in different ways, such as inflammatory responses or molecular receptor binding, which contributes to bacterial translocation across host BMEC monolayers (Al-Obaidi and Desa, 2018).

Castro-Garza et al. (2012) reported that bacterial cytotoxicity is dependent on strain virulence and host intracellular interaction (Castro-Garza et al., 2012). In our study, we found that *C. sakazakii* caused a cytotoxic response, including LDH release and caspase-3 activation. Lactate dehydrogenase (LDH), a stable cytoplasmic enzyme, is a marker for cytomembrane damage and cellular permeability increase. It was reported that *Pseudomonas aeruginosa* stimulated host cell LDH release, contributing to bacterial intracellular transcytosis (Elsahn et al., 2020). In this work, it was observed that LDH was rapidly released after *C. sakazakii* infection. Meanwhile, the pro-apoptotic caspase-3 activity and Annexin V-FITC positive apoptotic cells in infected HBMEC increased. These observations are similar to *Listeria monocytogenes* invasion into the host epithelial cells. *L. monocytogenes-*induced cell stress responses and apoptosis facilitated bacterial invasion and translocation in epithelial cells (Dos Santos et al., 2011). Inducing host cell apoptosis is also an essential pathway for *Shigella flexneri* invasion in hepatocytes and translocation in the Caco-2 monolayer barrier (Lima et al., 2013).

Additionally, cytotoxicity and apoptosis are closely associated with inflammatory responses. Previous research found that pro-inflammatory cytokines, like TNF-α, activated caspase-3 and cell apoptosis (Zhao et al., 2016). Interestingly, we observed an increase in mRNAs of TNF-α, IL-6, and caspase-3 activations in *C. sakazakii*-infected HBMEC. We speculated that pro-inflammatory cytokines might stimulate the activation of caspase-3 and subsequently induce HBMEC apoptosis, leading to *C. sakazakii* translocation. It was noted that *C. sakazakii* may directly induce late stage apoptosis in HBMEC. These results are similar to methylglyoxal-induced HBMEC injury and apoptosis (Zhou et al., 2015). It can be inferred that the bacteriotoxin released by *C. sakazakii* may induce apoptosis in HBMEC directly, following the disruption of HBEMC monolayers. Liu Q. et al. (2012) reported that *Cronobacter sakazakii* isolates induced the increase of intestinal epithelial cells monolayer permeability and cell apoptosis during bacterial translocation through intestinal barriers. Li et al. (2010) and Liu D. X. et al. (2012) justified *C. sakazakii* could stimulate cPLA(2)α-regulated Akt signaling pathway activation, PI3K-mediated actin filaments rearrangements, and cell apoptosis in HBMEC, which were required for *C. sakazakii* invasion in HBMEC.

Disruption of the BBB tight junction structure is one of the possible mechanisms for bacterial translocation. After exposure to *E. coli* for a long period of time, a widening of tight-junctions and formation of holes were observed in the Caco-2 monolayer, leading to a lower TEER and increased Dextran-FITC permeability (Yuan et al., 2020). In our study, *C. sakazakii* decreased the TEER and increased Dextran-FITC permeability. Similarly, *Pseudomonas aeruginosa* exoprotein induced mucosal barrier disruption evidenced by increased permeability of Dextran-FITC and decreased TEER after 4 h of challenge (Li et al., 2019). A reduction was observed in the protein levels of ZO-1 and Occludin among *C. sakazakii*-infected HBMEC. It was also proved that certain bacteria, like Group B *Streptococcus agalactiae*, disrupted the tight junction proteins claudin-5, Occludin, and ZO-1, which was necessary for bacterial translocation in BBB (Kim B. J. et al., 2017; Jiao et al., 2011). In a rat model, the reduced levels of ZO-1/Occludin increased the permeability of BBB and facilitated transcytosis (Wang et al., 2019). Taken together, *C. sakazakii* infection resulted in increased cellular permeability and disruption of the tight junction, which contributes to bacterial translocation across HBMEC monolayers.

In summary, *C. sakazakii* strains could invade and survive in HBMEC, and translocate across HBMEC monolayers. Endocytosis vesicles were observed in *C. sakazakii-* invaded HBMEC. *C. sakazakii* also induced inflammatory responses and apoptosis in HBMEC. Meanwhile, the permeability of HBMEC monolayers increased and the tight junction was disrupted after *C. sakazakii* infection. These findings reveal the possible transcellular and paracellular pathways for *C. sakazakii* ATCC 29544 to translocate across HBMEC monolayers. However, this *in vitro* study was performed mainly with one virulent strain and one cell line forming BBB. Future studies will test more *C. sakazakii* strains in *in vitro* and *in vivo* models to discover detailed mechanisms by which *C. sakazakii* translocates through HBMEC monolayers and BBB.

## Data Availability Statement

The raw data supporting the conclusions of this article will be made available by the authors, without undue reservation.

## Author Contributions

TJ and XX conceived and designed the experiments and wrote the manuscript. TJ, NG, and YD performed the experiments. XZ and JL analyzed the data. NG and XZ contributed reagents, materials, and analysis tools. All authors contributed to the article and approved the submitted version.

## Conflict of Interest

The authors declare that the research was conducted in the absence of any commercial or financial relationships that could be construed as a potential conflict of interest.
